# Tailored lighting intervention (TLI) for improving sleep-wake cycles in older adults living with dementia

**DOI:** 10.3389/fphys.2023.1290678

**Published:** 2023-12-18

**Authors:** Mariana G. Figueiro, David Pedler, Barbara Plitnick, Erick Zecena, Sophie Leahy

**Affiliations:** Department of Population Health Science and Policy, Light and Health Research Center, Icahn School of Medicine at Mount Sinai, New York, NY, United States

**Keywords:** Alzheimer’s disease and related dementias, assisted living, circadian stimulus, circadian system, light therapy, lighting systems, sleep-wake cycles, stimulus delivery

## Abstract

**Introduction:** Sleep disturbance is a hallmark of Alzheimer’s disease and related dementias, and caregiver stress caused by patients’ nighttime wandering, injuries, and agitation are frequently at the root of decisions to move them to assisted living facilities, where typically dim institutional lighting can further exacerbate their sleep problems. This study explored the effects of a circadian-effective lighting intervention on actigraphic sleep measures and subjective assessments of sleep disturbance, depression, and sleep-disturbed behaviors.

**Methods:** Fourteen older adult (≥60 years) participants (11 females, mean age = 84.1 [SD 8.9]), all diagnosed with moderate to severe dementia and sleep disturbance, were recruited from 3 assisted living and memory care facilities. Following a crossover, placebo-controlled design, 3 different lighting modes were used to deliver high levels of circadian stimulus to the participants’ eyes for two 8-week intervention periods in a counter balanced order with a 4-week washout between the study’s 2 conditions (dim light control vs. active intervention). Actigraphy and questionnaire data were collected over 7-day assessment periods that preceded (baseline weeks 1 and 9) and concluded (post-intervention week 9 and 22) the intervention periods. Actigraphic outcomes included sleep duration, sleep time, sleep efficiency, sleep start time, and sleep end time. Subjective assessments included the Cornell Scale for Depression in Dementia (CSDD), Pittsburgh Sleep Quality Index (PSQI), and Sleep Disorders Inventory (SDI) instruments.

**Results:** Under the active condition, sleep duration significantly (*p* = 0.018) increased and sleep start time significantly (*p* = 0.012) advanced after the intervention compared to baseline. Also under the active condition, PSQI (*p* = 0.012), CSDD (*p* = 0.007), Sleep Disorders Inventory frequency (*p* = 0.015), and SDI severity (*p* = 0.015) scores were significantly lower after the intervention compared to baseline.

**Discussion:** This study demonstrates that a circadian-effective lighting intervention delivering bright days and dark nights improves measures of sleep and mood in dementia patients living in controlled environments.

## 1 Introduction

Dementia is a presently incurable, progressive neurodegenerative disease for which there are very few effective treatments. Alzheimer’s disease (AD), the most common form of dementia, is the sixth-leading cause of death in the U.S. and the fifth-leading cause of death for those over the age of 65. With the population of that age cohort expected to more than double from 49 million in 2016 to 95 million in 2060 (Vespa et al., 2020), the number of Americans living with AD and related dementias (ADRD) is projected to reach 13.8 million by 2060 in the absence of treatments to diminish or cure the disease (Alzheimer’s Association, 2023). More than 70% of people with the disease live at home, and more than 80% of their required care is provided by unpaid family members, friends, and neighbors (Alzheimer’s Association, 2023).

As the disease progresses, families are often compelled to relocate their loved ones to assisted living facilities, a move often precipitated by caregiver stress and fatigue caused by nighttime wandering, injuries, and agitation associated with the individuals’ disturbed circadian sleep-wake cycles. In addition to sleep disturbances associated with emotional stress resulting from relocation, especially when the move is involuntary (Mallick and Whipple, 2000; Castle, 2001), individuals’ sleep can be even further disrupted by the typically constant dim lighting found in nursing homes and assisted living facilities. Because such lighting lacking a robust light-dark pattern can be inadequate for stimulating residents’ circadian systems and promoting entertainment (Ancoli-Israel and Kripke, 1989; Ancoli-Israel et al., 1997b; Shochat et al., 2000), light therapy that provides robust daytime light exposures shows great promise as a nonpharmacological treatment for residents in those settings. Research has shown that lighting interventions can regulate the timing of sleep, improve sleep quality and cognition, and reduce symptoms of depression and nighttime agitation in older adults, both with and without AD/ADRD (Hanford and Figueiro, 2013; Kolberg et al., 2021a; Baandrup and Jennum, 2021; Kolberg et al., 2021b; Cibeira et al., 2021; Cremascoli et al., 2022; Kim et al., 2022).

This study proceeds from preliminary data showing that a lighting intervention that is carefully designed and delivered to maximally affect the human circadian system can improve objective and subjective measures of sleep and subjectively assessed measures of mood and behavior in ADRD patients living in controlled environments. Our premise is that patients live in continuous dim light and that a circadian-effective lighting intervention tuned to the spectral sensitivity of the human circadian system can exert significant impacts on sleep, mood, and behavior in this population. Specifically, we hypothesized that a tailored lighting intervention (TLI) would be associated with actigraphic measures of significantly earlier bed and wake times, longer sleep episodes, and more consolidated rest-activity patterns compared to the baseline condition. Furthermore, we hypothesized that the TLI would be associated with significantly lower scores on subjective assessments of sleep disturbance, depression, and sleep-disturbed behaviors. Based on our previous studies, our primary outcome for the present study was Pittsburgh Sleep Quality Index (PSQI) scores.

## 2 Materials and methods

### 2.1 Participant selection

The sample size for this study was calculated according to one of our previous studies examining actigraphy measurements and questionnaire scores before and after customized self-luminous light table interventions (Figueiro et al., 2016). Based on this *a priori* work, we determined an effect size between pre- and post-intervention to be 1.1 for questionnaire scores (based on our primary outcome, mean [SD] PSQI) and 0.6 for sleep actigraphy measurements (based on mean [SD] sleep efficiency). The required sample size to see a significant difference between pre- and post-intervention (in the active condition) was calculated utilizing these effect sizes and the following parameters: two-sided (matched pairs) test for a non-parametric analysis, 0.05 alpha level, and 80% power. The suggested enrollment for the study was 9 subjects based on questionnaire scores (primary outcome) and 26 subjects based on actigraphy sleep measurements. While we fulfilled the needed sample size for questionnaire score comparisons, there were missing subjects due to lack of sufficient pool of participants who met the inclusion/exclusion criteria, so we did not meet the sample size suggestion for actigraphy comparisons. However, even so, we were able to detect significant differences between pre-and post-interventions (in the active condition) for the sleep duration and sleep start time outcomes.

Fourteen participants (11 females, mean age = 84.1 [SD 8.9]) were recruited from 3 assisted living and memory care facilities managed by the Salmon Health and Retirement Group in Westborough, MA (5 participants), Northborough, MA (4 participants), and Medway, MA (5 participants), United States. The facilities were under the same administration, so day-night schedules and the overall environment were the same for all 3 facilities. Although 3 of the participants withdrew before completing both arms of the study (one passed away, one broke a hip and transferred out of the facility, and one withdrew because she did not like the lights), all their available data were included in the analyses, as detailed in the statistical analyses section. For inclusion in the study, participants were to be aged 60 years and older, diagnosed with moderate to severe AD/ADRD (indicated by a Global Deterioration Scale score of 4.0–6.0) (Reisberg et al., 1982), and documented to have sleep disturbance (indicated by a score >5 on the Pittsburgh Sleep Quality Index [PSQI]) (Buysse et al., 1991). The mean Brief Cognitive Rating Scale (BCRS) score was 4.83 [SD 1.01] and the mean Global Deterioration Scale (GDS) was 4.89 [SD 1.1]. In the BCRS scale, a score of 4 indicates moderate impairment and a score of 5 indicates moderately severe impairment. As for the GDS, a score of 4 indicates moderate cognitive decline (mild dementia) and a score of 5 indicates moderately severe cognitive decline (moderate dementia).

Participants using any sleep medication (e.g., melatonin, Trazadone, and any benzodiazepines) were excluded, as were those diagnosed with obstructing cataracts, macular degeneration, and blindness. Informed consent was obtained from the participants and/or their caregivers. The study was conducted in accordance with the Declaration of Helsinki (World Medical Association, 2000) and was approved by the Institutional Review Boards at Rensselaer Polytechnic Institute and the Icahn School of Medicine at Mount Sinai.

### 2.2 Lighting interventions

The specification for the study’s circadian-effective lighting intervention is the circadian stimulus (CS) metric (Rea et al., 2021a; Rea et al., 2021b), which is based on published recommendations (Underwriters Laboratories, 2019) and supported by previous field studies showing that participants from various populations (including older adults living with all stages of AD/ADRD) who received daytime CS levels ≥0.3 at the eyes experienced improved sleep and reduced depression (Young et al., 2015; Figueiro et al., 2016; Figueiro and Rea, 2016; Figueiro et al., 2017; Figueiro et al., 2019a; Figueiro et al., 2019b; Figueiro et al., 2020; Zhou et al., 2022). Circadian-effective light (CL_A_) is based upon the spectral sensitivity of the human circadian system (Rea et al., 2005; Rea et al., 2012). All 3 photoreceptor types in the retina (i.e., rods, cones and the intrinsically photosensitive retinal ganglion cells, ipRGCs) contribute in some way to CL_A_ spectral sensitivity. Perhaps most importantly, CL_A_ values are determined by how those photoreceptors’ signals are combined by the neurons in the proximal retina before exiting the eye. Those neurons also determine the magnitude of the photic stimulus exiting the eye before reaching the SCN. Circadian stimulus (CS) magnitudes (Eq. 1) are determined from the CL_A_ values through post-processing. Low CL_A_ values are not converted into CS magnitudes if they are, according to the model, below threshold. Above the modelled threshold, CL_A_ values are transformed into CS values until they reach a point of diminishing return where still higher CL_A_ values have no incremental effect on CS. Thus, CS models both the spectral and absolute sensitivities of the phototransduction processes in the retina that stimulate the SCN (Rea et al., 2010; Rea et al., 2021a; Rea et al., 2021b).

C S =0.7 ∗ 1− 11+CLA355.71.1026 (1)

Values of CS are, by definition, equal to the predicted levels of light-induced nocturnal melatonin suppression after 1 h of exposure, from threshold to saturation (Rea et al., 2010). Although derived from nocturnal melatonin suppression, CS is an instantaneous characterization of light as the photic stimulus to the SCN.

In the present study, 3 lighting devices delivering high levels (≥0.3) of CS to the participants’ eyes were implemented: 1) custom-built, self-luminous light tables (*n* = 2) installed in the dining/community room at the study’s Westborough site, 2) custom-built, self-luminous cafeteria-style light trays (*n* = 4) placed on conventional dining tables at the Northborough site, and 3) redesigned color-tunable ambient room lighting installed in a dining/community room at the Medway site.

Five participants were exposed to the light table intervention, 4 participants were exposed to the light tray intervention, and 5 participants were exposed to the ambient room lighting intervention. All participants experienced either an active or control intervention for 8 weeks and then crossed over for exposure to the opposite intervention for an additional 8 weeks following a 4-week washout period (Section 2.4). Laboratory measurements of the light tables and trays were collected via spectroradiometer (model BTS 256, Gigahertz-Optik, Tuerkenfeld, DE), photometer (model X91, Gigahertz-Optik), and illuminance meter (model LS-100, Konica Minolta, Ramsey, NJ, United States). The specifications for the active and control interventions for each device are described below. Commission Internationale de l'Éclairage (2018) compliant alpha-opic values calculated for the 3 lighting intervention devices, following the system for metrology of optical radiation for ipRGC (intrinsically photosensitive retinal ganglion cells)-influenced responses to light, are provided in Supplementary Table S1.

#### 2.2.1 Light tables

**FIGURE 1:**
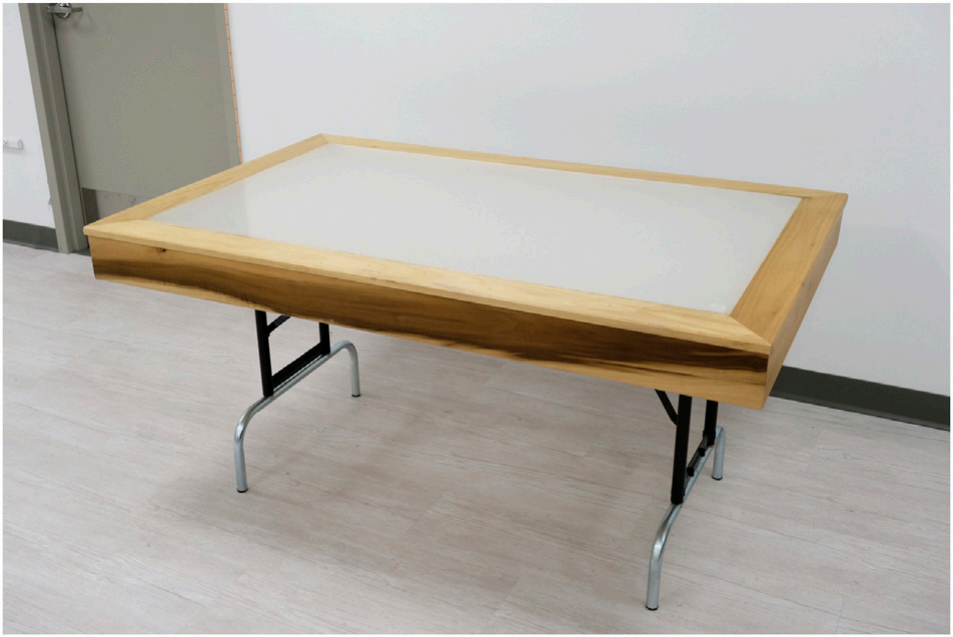
The light table intervention device.

The custom-built light tables (XtraLight, Houston, TX, United States) employed in the intervention at the Westborough site measure (l × w × h) 156 cm × 99 cm × 13 cm, with a luminous area on the tabletop measuring 137 cm × 79 cm. When the 64 cm long legs are extended the tabletop sits 76 cm above the floor (Figure 1). The light is provided by 13 light-emitting diode (LED) strips measuring (l × w) 61 cm × 6 cm (model BB0040, ver. 001.1, XtraLight) recessed 9.5 cm beneath the tabletop and covered by a 1 cm thick diffused acrylic lens that serves as the table’s durable work surface.

The participants were encouraged to sit at the 2 light tables employed at the Westborough site whenever they were using the dining/community room over the course of the study. As measured in the laboratory at eye level when seated with a single dinner plate occluding the table’s surface, the active intervention delivered 1887 lx and a CS of 0.625. Unfortunately, the site nurses changed the light tables’ correlated color temperature for the control condition but neglected to dim it as instructed. Thus, because the control condition was still delivering high CS values, the data for these subjects were removed from the analysis (Section 2.5).

#### 2.2.2 Light trays

**FIGURE 2:**
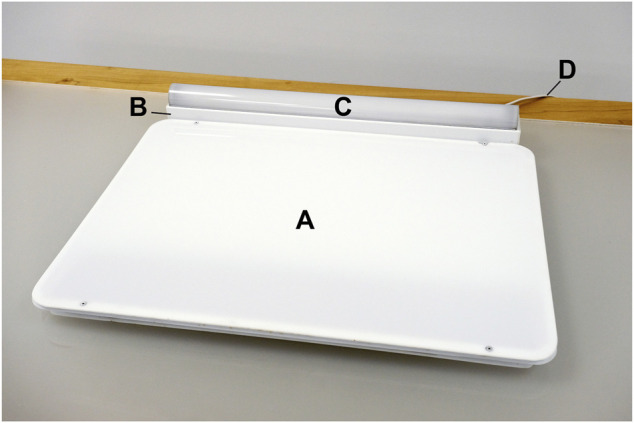
The light tray intervention device, showing the position of the LED-lit dining/work surface **(A)**, power supply **(B)**, LED strip **(C)**, and power cord **(D)**.

The light trays employed at the Northborough site were custom built by the LHRC to serve as light-emitting dining/work surfaces and fashioned after a conventional cafeteria tray. The device measures (l × w × h) 64 cm × 53 cm × 4 cm, including the 61 cm × 7.6 cm × 6.4 cm power supply/controller traversing the distal length of the tray, which is topped by a 60 cm × 3 cm × 3 cm LED strip covered with quarter-round diffuser (Figure 2). The participants were assigned to seats facing the tabletop light trays for breakfast and lunch over the course of the study. As measured in the laboratory at eye level when seated with a single dinner plate occluding the tray’s surface, the active intervention delivered 602 lx and a CS of 0.456 and the control condition delivered 65 lx and a CS = 0.064. As the trays could deliver only a single condition, they were alternated between the active intervention and the control.

#### 2.2.3 Ambient room lighting

**FIGURE 3:**
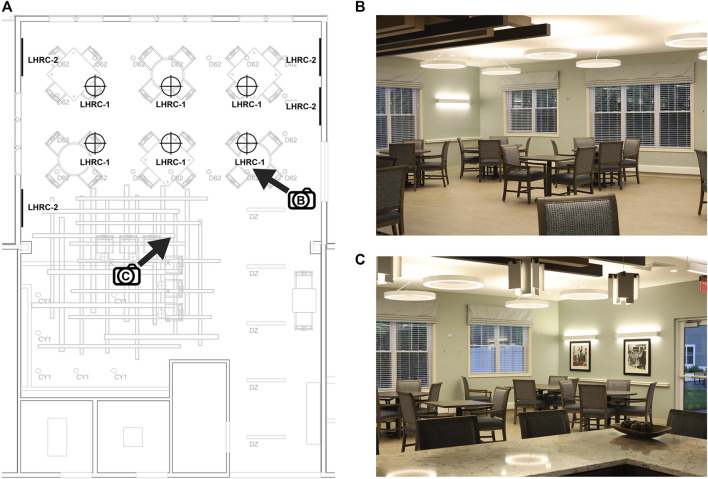
The lighting retrofit (LHRC-1, LHRC-2) at the Medway site **(A)** and two views of the community/dining room **(B,C)** indicated by the directional arrows in **(A)**. The pre-existing lighting is shown in gray and represented by the codes CY1 (3500 K cylindrical pendant, Acuity Brands, Atlanta, GA, United States), D62 (3000 K recessed downlight, Spectrum Lighting, Fall River, MA, United States), DZ (3500 K direct/indirect linear pendant, Mark Architectural Lighting [Acuity Brands]), and X (2700 K decorative pendant, Lightology, Chicago, IL, United States).

The retrofit of the dining/community room lighting at the Medway site was performed with assistance from the researchers. Before the retrofit, the design employed several lighting fixtures sourced from a variety of manufacturers (Figure 3). In the area that received the retrofitted lighting, the mean illuminance measured at the eyes for occupants seated at the tables was 307 lx and the mean CS value was 0.28. The retrofit employed six 30-in diameter dimmable, direct/indirect circular (5-in lens aperture) ring pendant lighting fixtures (3000 K; Sketch, Axis Lighting, Lasalle, QC, CA; LHRC-1) suspended over the dining/community room’s six tables and four 4-ft linear dimmable, direct/indirect (2-in lens aperture) (3000 K; Beam 2, Axis Lighting; LHRC-2) fixtures mounted on adjacent, opposing walls (see Figure 3). The retrofitted system was driven by a programmable power/relay pack and on-screen controls (Acuity Brands, Conyers, GA, United States) to deliver a CS of 0.4 from 07:00 to 17:00, gradually transitioning (from 17:00 to 18:00) to a CS < 0.1 from 18:00 to bedtime.

Measurements in the Medway site community room were performed with spectroradiometer to confirm the targeted light levels. Average vertical illuminance (measured at eye level when seated at the table) was 520 lx (mean CS = 0.37) for the intervention lighting and 135 lx (CS = 0.1) for the control condition. Because the intervention and control conditions involved the ambient room lighting and could not be crafted to individual seating locations, all participants were exposed to the high CS daytime schedule and low CS daytime schedule during the same counterbalanced 8-week intervention periods.

### 2.3 Study outcomes

#### 2.3.1 Actigraphy

Participants were asked to wear an actigraph (Actiwatch-2, Philips Respironics, Murrysville, PA, United States) for 7 days during the study’s assessment weeks (Section 2.4). The size of a small watch, the device was worn on each participant’s nondominant wrist. Actigraphy has been demonstrated in prior studies to be well tolerated by older adults with ADRD (Ancoli-Israel et al., 1997a; David et al., 2010; Solomons and Chatterjee, 2016; Smith et al., 2018), and it is strongly correlated with polysomnography (PSG) and sleep logs (Kushida et al., 2001; Camargos et al., 2013; Kosmadopoulos et al., 2014). The actigraph data were processed using the device manufacturer’s software (Actiware version 6.0.9, Philips Respironics) to calculate the following sleep parameters: sleep duration (i.e., total minutes between sleep start time and sleep end time), sleep time (the sum of 1-min epochs scored as sleep, in minutes), sleep efficiency (sleep time as a percentage of sleep duration), sleep start time (in hours past the preceding midnight [24:00]), and sleep end time (in hours past the preceding midnight [24:00]).

In addition, using the actigraphy data, we calculated 2 additional metrics: 1) interdaily stability (IS), a ratio indicating the strength of coupling between the light-dark cycle and rest-activity rhythm over a 24-h period; and 2) intradaily variability (IV), an indication of the fragmentation of the rest-activity rhythm (i.e., the frequency of the transitions between rest and activity).

#### 2.3.2 Questionnaires

Three questionnaires were used to measure depression, sleep quality, and caregiver-assessed burden of participants’ sleep-disturbed behaviors.

The Pittsburgh Sleep Quality Index (PSQI) (Buysse et al., 1989; Buysse et al., 1991) was used to measure sleep quality via the assessment of 19 items that generate 7 component scores (i.e., subjective sleep quality, sleep latency, sleep duration, habitual sleep efficiency, sleep disturbances, use of sleep medication, and daytime dysfunction). The sum of the 7 component scores yields a single global score with values > 5 indicating sleep disturbances. The PSQI is a well validated scale and has been used previously in patients with AD (Figueiro et al., 2014; Durães et al., 2023).

The Cornell Scale for Depression in Dementia (CSDD) (Alexopolous et al., 1988; Korner et al., 2006) is a 19-item tool designed to rate symptoms of depression in persons with dementia. It was used to evaluate the presence and extent of mood-related signs (i.e., anxiety, sadness, and irritability); behavioral disturbances (i.e., agitation and loss of interest); physical signs (i.e., loss of appetite and weight loss); cyclic functions (i.e., mood variation, sleep quality); and ideational disturbances. The items are scored 0–3 points and a total score >12 indicates probable depression.

The Sleep Disorders Inventory (SDI) (Tractenberg et al., 2003) is an expanded version of one item from the Neuropsychiatric Inventory that assesses the frequency, severity, and caregiver burden (named “distress” in the instrument) of participants’ sleep-disturbed behaviors. The SDI is a simple and short instrument that can be administered multiple times during a study protocol. The SDI measures the occurrence of disturbances during the preceding week using variable-length numeric scales with scores ranging from zero to no greater than 5 points and higher scores representing greater frequency. Research has demonstrated the validity of using caregiver responses to SDI sleep questions as a nonredundant, independent instrument in sleep research, showing good correlation with quantitative sleep measurements (Tractenberg et al., 2003).

### 2.4 Study protocol

**FIGURE 4:**
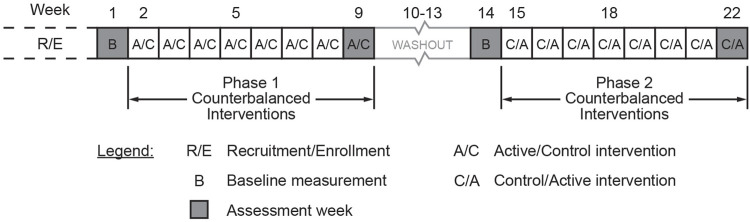
The study protocol.

Each lighting intervention was presented for 8 weeks in a counter balanced order with a 4-week washout between conditions (Figure 4). All data points were collected during the baseline (week 1) and repeated at weeks 9 (post intervention), 14 (baseline post washout) and 22 (post intervention). Data collection at the Medway site occurred between January and June 2022, and data collection at the Westborough and Northborough sites occurred between July and December 2022. It should be noted that activities schedules (e.g., eating, sleeping, recreational activities) were the same in all 3 facilities, as they were all under the same management.

Once consent was obtained, baseline screening was administered by the research coordinator or study RN using the CSDD, PSQI, and SDI questionnaires. The questionnaires took approximately 10 min to complete. If the participants were unable to self-complete these questionnaires, we worked with the charge nurse or social worker at the facility to have a staff member most familiar with the participants’ sleep and behavior complete the questionnaires. We asked that the same staff member complete the questionnaires each time during the protocol. Participants were asked to wear a wrist actigraph for 1 week to obtain baseline data on rest/activity patterns and sleep quality. Following this initial baseline collection, the lighting intervention, either active or placebo control, was energized. The lighting intervention (active or control) remained in place for 8 weeks. Each subject participated in the study for a total of 22 weeks. All assessments were completed during the baseline (week 1) and repeated at weeks 9 (post-intervention Phase 1), 14 (baseline Phase 2), and 22 (post-intervention Phase 2).

### 2.5 Statistical analysis

Given that the 3 light delivery devices were designed to deliver a CS of at least 0.3 in the active condition and a CS of <0.1 in the control condition, it follows that no statistically significant differences were observed between the results obtained from participant exposures to those devices (Supplementary Material; Supplementary Figures S1, S2). The data recorded for all participants were therefore combined in the statistical analyses.

A counterbalanced design analysis ideally should include a statistical examination of any carryover effect between the 2 intervention periods. However, because the participants using the light tables who experienced the control condition first had missing data due to noncompliance with the protocol, we were unable to account for condition order and thereby determine whether there was a carryover effect. Nonetheless, our previous research (Figueiro et al., 2019b) suggests that there would have been a minimal carryover effect, if any, after the 4-week washout period employed in the present study (Section 4).

Statistical analysis was performed using SPSS Statistics (version 28.0, IBM, Armonk, NY, United States). A non-parametric Wilcoxon matched pairs signed ranks test was used to compare actigraphy data (i.e., sleep duration, sleep time, sleep efficiency, sleep start time, and sleep end time) and questionnaire data (i.e., PSQI, CSDD, SDI frequency, SDI severity, and SDI distress) between baseline and intervention. The analysis was performed separately for the active and control conditions, and statistical significance was accepted at *p* < 0.05.

Data from 14 participants were employed in the statistical analysis, including those from 10 participants who experienced the active intervention first and from 4 participants who experienced the control condition first. For conditions with available baseline measurements but missing intervention measurements, intervention data (actigraphy, *n* = 3 in the active condition; questionnaires, n = 1 in the active condition, *n* = 2 in the control condition) were imputed by the last observation carried forward from baseline. Statistical analysis was performed for 7 control condition subjects and 11 active condition subjects in the actigraphy outcomes, and 12 control condition subjects and 12 active condition subjects in the questionnaire outcomes. The participant breakdown of baseline and intervention data available for analysis in the actigraphy and questionnaire outcomes is shown in Table 1 by condition order.

**TABLE 1:** Participant breakdown of baseline and intervention data available for analysis in the actigraphy and questionnaire outcomes.

Data available for analysis	Analyses	Number or participants by condition order	
		Active–control (*n* = 10)	Control–active (*n* = 4)
Active condition, baseline	Actigraphy	10	1
	Questionnaires	10	2
Active condition, intervention	Actigraphy	10 (3 imputed)	1
	Questionnaires	10 (1 imputed)	2
Control condition, baseline	Actigraphy	7	1
	Questionnaires	8	4
Control condition, intervention	Actigraphy	7	1
	Questionnaires	8 (2 imputed)	4

## 3 Results

### 3.1 Actigraphy

**FIGURE 5:**
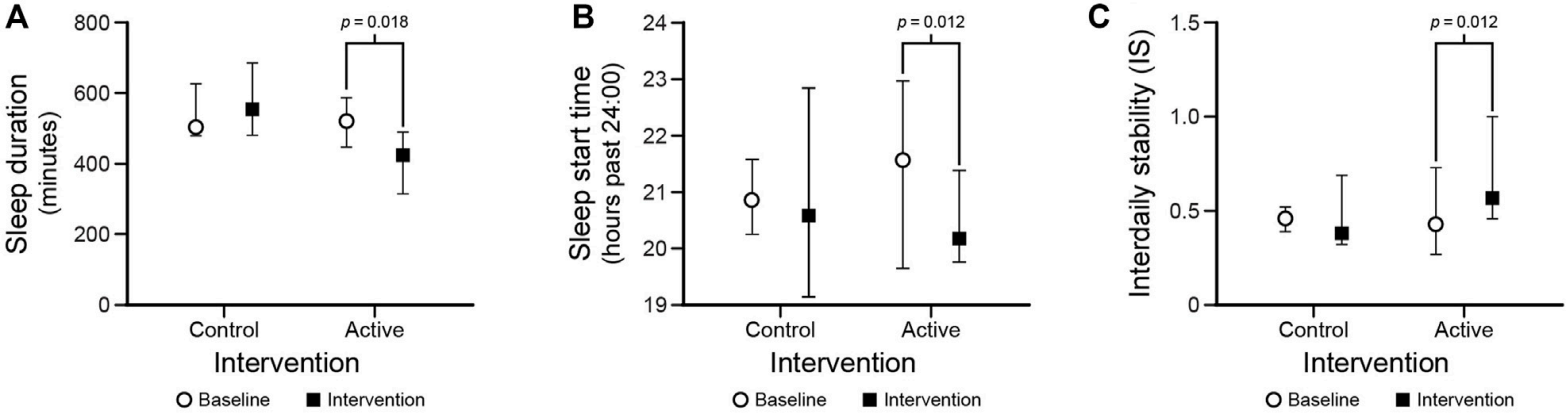
Statistically significant results for the sleep duration **(A)** and sleep start time **(B)**, and interdaily stability **(C)** outcomes. The circles and squares represent median values and the bars represent IQRs.

Median (interquartile range [IQR]) actigraphy values are presented by outcome in Table 2 (all results) and Figure 5 (statistically significant results only). Under the control condition, there were no significant differences between baseline and the intervention for sleep duration, sleep time, sleep efficiency, sleep start time and sleep end time (*p* > 0.31).

**TABLE 2:** Summary of actigraphy analysis results.

Outcome	Control (*N* = 8)			Active (*N* = 11)		
	Baseline	Intervention	*p*	Baseline	Intervention	*p*
Sleep duration, mean (IQR) [minutes]	504 (479, 626.27)	554.34 (480.25, 684.5)	0.779	520 (447,587)	584 (533,639)	0.018*
Sleep time, mean (IQR) [minutes]	426.5 (268.75, 518.5)	435.28 (340.75,476)	0.779	424 (314, 489)	456 (383,563)	0.237
Sleep efficiency, mean (IQR) [%]	74.5 (54.25, 89.08)	71.56 (59.75, 83)	0.498	81 (66, 86)	79 (65,88)	0.233
Sleep start time, mean (IQR) [hours past 24:00]	20.88 (20.28, 21.60)	20.59 (19.17, 22.84)	0.889	21.58 (19.67, 22.97)	20.20 (19.78, 21.40)	0.012*
Sleep end time, mean (IQR) [hours past 24:00]	6.19 (5.35, 7.18)	6.79 (6.06, 7.16)	0.575	6.42 (5.8, 7.17)	6.33 (5.48,6.70)	0.575
IS, mean (IQR)	0.46 (0.39,0.52)	0.385 (0.323, 0.690)	0.735	0.43 (0.27,0.73)	0.57 (0.46,1.0)	0.012*
IV, mean (IQR)	1.05 (0.75,1.72)	1.12 (1.07,1.68)	0.108	0.85 (0.64,1.14)	1.32 (0.53, 1.53)	0.092

Statistically significant values are shown in bold.

Under the active condition, sleep duration increased after the intervention compared to baseline (*p* = 0.018). Additionally, sleep start time was earlier after the intervention compared to baseline (*p* = 0.012). There were no differences between baseline and the intervention for sleep time, sleep efficiency, or sleep end time (*p* > 0.05).

Under the active condition, IS significantly increased after the intervention compared to baseline (*p* = 0.012). There were no significant differences between baseline and intervention for IS under the control condition. Moreover, there were no significant differences between baseline and intervention for IV in the control and the active conditions.

It should be noted that there were no significant differences between the baseline scores under active and control conditions for all the actigraphy measures.

### 3.2 Questionnaires

**FIGURE 6:**
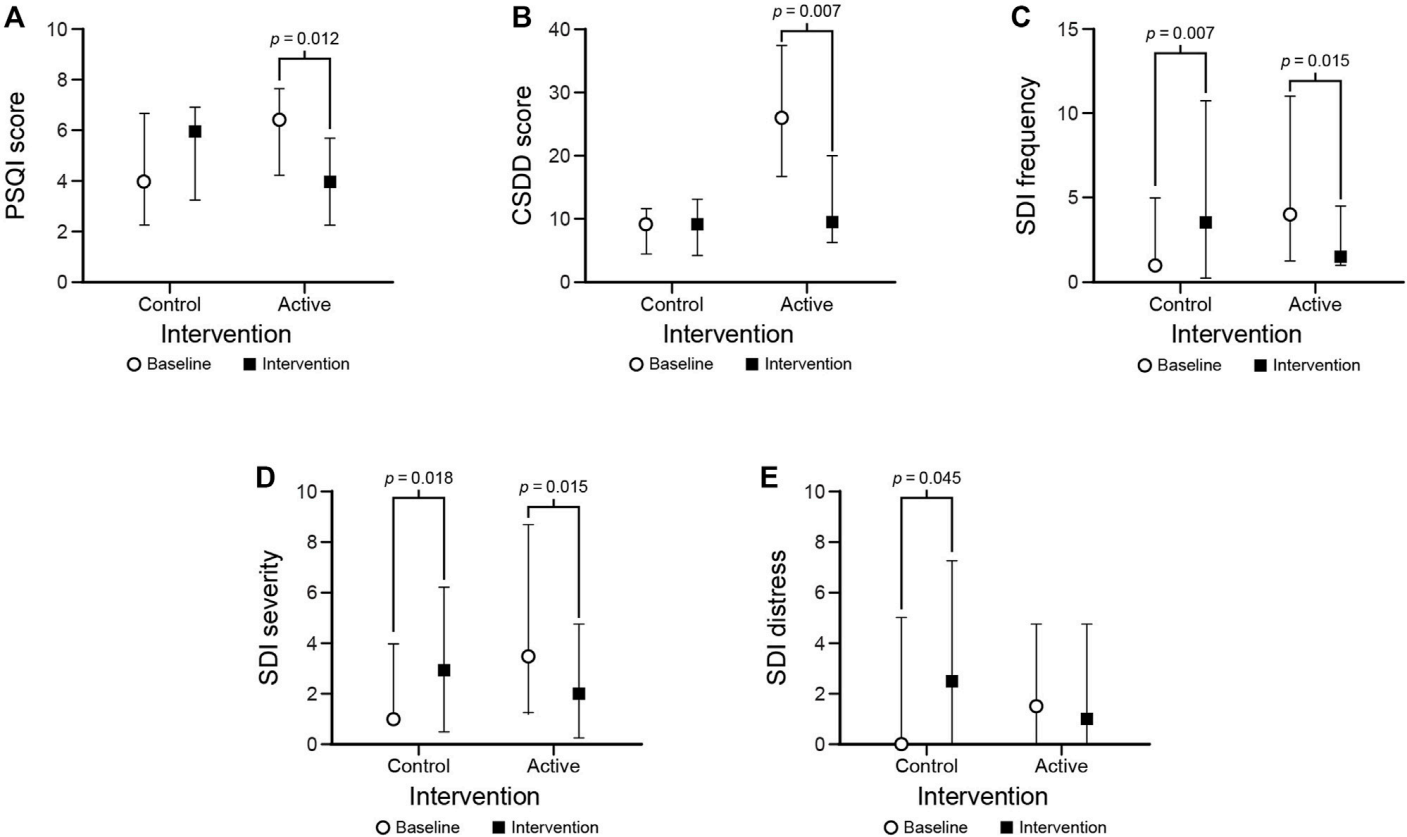
Statistically significant results for the PSQI **(A)**, CSDD **(B)**, SDI frequency **(C)**, SDI severity **(D)**, and SDI distress **(E)** outcomes. The circles and squares represent median scores and the bars represent IQRs.

Median (IQR) questionnaire scores are presented in Table 3 (all results) and Figure 6 (statistically significant results only). PSQI scores decreased after the intervention compared to baseline under the active condition but not under the control condition (*p* = 0.012 and *p* = 0.232, respectively). CSDD scores were lower after the intervention compared to baseline under the active condition but not under the control condition (*p* = 0.007 and *p* = 0.918, respectively). SDI frequency and SDI severity scores decreased after the intervention compared to baseline under the active condition (*p* = 0.015); they also both increased after the intervention compared to baseline under the control condition (*p* < 0.02). Under the control condition, SDI distress scores increased after the intervention (*p* = 0.045) but there was no significant difference in the active condition.

**TABLE 3:** Summary of questionnaires analysis results.

Outcome	Control (*N* = 12)			Active (*N* = 12)		
	Baseline	Intervention	*p*	Baseline	Intervention	*p*
PSQI, mean (IQR)	4 (2.25,6.75)	6 (3.25,7)	0.232	6.5 (4.25,7.75)	4 (2.25,5.75)	0.012*
CSDD, mean (IQR)	9 (4.25,11.5)	9 (4,13)	0.918	36 (16.75,37.5)	9.5 (6.25,20)	0.007*
SDI frequency, mean (IQR)	1 (1,5)	3.5 (0.25,10.75)	0.007*	4 (1.25,11)	1.5 (1,4.5)	0.015*
SDI severity, mean (IQR)	1 (1,4)	3 (0.5,6.25)	0.018*	3.5 (1.25,8.75)	2 (0.25,4.75)	0.015*
SDI distress, mean (IQR)	0 (0,5)	2.5 (0,7.25)	0.045*	1.5 (0,4.75)	1 (0,4.75)	0.343

Statistically significant values are shown in bold.

It should be noted that there were no significant differences between the baseline scores under the active and control conditions for all but one (CSDD) of the questionnaire measures. The CSDD score at baseline under the intervention condition was significantly higher (*p* < 0.001) than under the control condition. If using the score obtained during the baseline under the control condition and comparing it to the score obtained after the intervention in the active condition, the statistical significance is lost (*p* > 0.05).

## 4 Discussion

This crossover, placebo-controlled study confirms our previous studies showing that circadian-effective light (characterized as a CS > 0.3) delivered to ADRD patients living in controlled environments can improve measures of objective sleep as well as subjective sleep and mood (Figueiro et al., 2008; Hanford and Figueiro, 2013; Figueiro et al., 2014; Figueiro et al., 2019b; Figueiro et al., 2020). The present study extends the research literature in 2 ways. First, it adds to a small study performed by Figueiro et al. (2016) showing that the same light table delivering high CS to those with dementia and living in a nursing home improved sleep, mood, and behavior. Second, it demonstrates, for the first time, that a lighting system designed to deliver ambient circadian-effective light in a common area in an assisted living center is effective for improving sleep and mood in ADRD patients.

Several recent studies have investigated the impact of a dynamic lighting system on sleep and circadian parameters as well as mood in older adults with and without dementia. Baandrup and Jennum (2021) recruited 24 older nursing home residents with cognitive deficiencies, installing a dynamic lighting intervention in their private apartments within the facility. Residents in the experimental group (n = 12) were exposed to the intervention for 4 consecutive weeks while the control group (n = 12) was exposed to conventional lighting for the same duration. Participants in the experimental group experienced reduced intradaily variability (IV), which indicates the rest–activity rhythm’s fragmentation (i.e., the frequency and extent of transitions between rest and activity). These results are not consistent with the present results as we were able to show a significant increase in IS but not a significant decrease in IV. Because the small sample size, the study’s between-subjects design, and the short duration of the intervention may have reduced the effectiveness of the intervention, the authors recommended a longer intervention period with a greater number of subjects to further investigate the effectiveness of the dynamic lighting system.

Wahnschaffe et al. (2017) installed dynamic lighting in the common room of a nursing home and programmed the system to produce high illuminance with higher short-wavelength content during the day and lower illuminance and less short-wavelength content during the evening. Agitation was assessed using the Cohen-Mansfield Agitation Index (CMAI) (Cohen-Mansfield et al., 1989) before and after the lighting intervention in 15 residents with dementia. Participants continuously wore actigraphs on their wrists for 6 months and CMAI data (pre- and post-intervention) were analyzed using the Wilcoxon matched pairs method (the same statistical approach used in the present study). The authors showed that CMAI sum-scores—mainly pertaining to non-physically aggressive behavior—were significantly reduced with the intervention from 30.2 (SD 5.1) to 27.9 (SD 2.6) (*N* = 12; *p* < 0.05). Results from the rest-activity analysis did not show differences of circadian amplitude and other circadian variables before and after the lighting installation. Consistent with the present results, Cremascoli et al. (2022) delivered a light therapy treatment tailored to the participants’ circadian phase as measured by dim light melatonin onset (DLMO). They showed a light-induced phase shift in DLMO consistent with the circadian phase response curve to light exposure, resulting in a reduced phase angle between DLMO and sleep start time as well as an improvement in subjective sleep quality (PSQI). They did not observe, however, any changes in actigraphy measures, while we observed an increase in sleep duration and an earlier sleep start time.

Our study, however, showed a positive impact from increasing the intervention’s light level during the day—*without* varying the light’s spectral properties—and then lowering light level to limit circadian stimulation prior to the participants’ bedtimes. Our results are consistent with those from Van Someren et al. (1997), who found that increased illumination in the living environment of older adults with ADRD increased the stability of their rest-activity rhythm following a 4-week treatment. Actigraphy data from that study showed improvements in participants’ IS, which is the strength of coupling of the rhythm to the supposed stable environmental *zeitgebers*, and IV, which is an indication of the fragmentation of the rest-activity rhythm (i.e., the frequency of the transitions between rest and activity). The present results partially corroborate with those in that we observed a significant increase in IS after the active intervention, but not after the control intervention. Thus, bright days and dark nights led to a more consolidated rest-activity pattern and better sleep.

Three studies administering high light levels (2,500–10,000 lx) to AD/ADRD patients for 2 h in the morning over periods ranging from 10 days to 4 weeks resulted in significantly longer sleep durations (Lyketosos et al., 1999), greater consolidation of sleep, (Yamadera et al., 1996; Ancoli-Israel et al., 2003), and decreased incidence of nighttime waking and daytime sleep (Yamadera et al., 1996). Sloane et al. (2007) showed a significant improvement in nighttime sleep from exposure to morning or all-day bright light, with greater improvement occurring among persons with severe dementia. Examining the effects of morning versus evening bright light on institutionalized persons living with AD, Dowling and others (Dowling et al., 2005a; Dowling et al., 2005b) found no significant differences in actigraphy-based measures of nighttime sleep or daytime waking. They did, however, find that participants experiencing the bright light intervention at either time evidenced a significantly more stable rest-activity rhythm acrophase over the 10-week treatment period (compared to the controls), with participants who had the most impaired rest-activity rhythm responding significantly and positively. In the present study, the light table and ambient room lighting TLI modes were available all day and were left unattended, so participants could have received the lights all day or just part of the day. Regardless, our results show that exposure to higher light levels at any time of the day leads to better nighttime sleep and mood.

This study has limitations. First, the sample size is small, although it is strengthened by its crossover, within-subjects design. The small sample size was due to the COVID-19 pandemic and some families were hesitant to allow their family members into the study. The counterbalancing of the experimental conditions (active versus control) was also incomplete and some degree of carryover effect may have influenced the results, although previous research from our lab has shown that a 4-week washout period is sufficient (Figueiro et al., 2019b). The light delivery systems differ for the various sites used in the study, but using CS as the rectifying variable highlights a strength rather than a limitation in this study; namely, that carefully specified circadian-effective light can be delivered using various modalities. Indeed, this approach can increase compliance—which is always a challenge in field settings like assisted living facilities—and improve effectiveness of the lighting intervention. We did not have information about participants’ dementia subtypes, so it was not possible to determine whether a certain dementia subtype (e.g., frontotemporal degeneration) was more sensitive to light treatment than others. Similarly, our study included a larger number of females than males, largely due to a high prevalence of dementia in females, and it is not yet established whether light sensitivity varies between males and females. Future studies should be designed and powered to investigate differences between dementia type and sex.

In summary, the present study extends findings from previously published studies demonstrating that a circadian-effective lighting intervention delivering bright days and dark nights improves sleep and mood in ADRD patients in controlled environments.

## Data Availability

The raw data supporting the conclusion of this article will be made available by the authors, without undue reservation.
