# Supplementary material for: Tailored lighting intervention (TLI) for improving sleep-wake cycles in older adults living with dementia
Source: Front Physiol. 2023 Dec 18;14:1290678. doi: 10.3389/fphys.2023.1290678 (PMC10759231; doi:10.3389/fphys.2023.1290678)
Supplement: Supplementary file 1 [file DataSheet1.pdf]

Supplementary Material

1 Measurements following the Commission Internationale de l'Éclairage (CIE) system for metrology of optical radiation for ipRGC (intrinsically photosensitive retinal ganglion cells)-influenced responses to light

Supplementary Table S1. Alpha-opic values calculated for the 3 lighting intervention devices employed in this study.

Device	CIE alpha-opic value	Active ($W.m^{-2}$)	Control ($W.m^{-2}$)
Light table	CIE S-cone irradiance	1.40	0.13
	CIE M-cone irradiance	2.64	0.80
	CIE L-cone irradiance	3.04	1.19
	CIE rhodopic irradiance	2.36	0.50
	CIE melanopic irradiance	2.05	0.37
Light tray	CIE S-cone irradiance	0.35	0.01
	CIE M-cone irradiance	0.81	0.07
	CIE L-cone irradiance	0.97	0.11
	CIE rhodopic irradiance	0.69	0.05
	CIE melanopic irradiance	0.59	0.04
Ambient room lighting	CIE S-cone irradiance	0.13	0.02
	CIE M-cone irradiance	0.61	0.13
	CIE L-cone irradiance	0.86	0.19
	CIE rhodopic irradiance	0.41	0.08
	CIE melanopic irradiance	0.32	0.06

2 Supplementary Results: Statistical Analysis of Differences Between the Three Lighting Intervention Devices for Study Outcomes

Supplementary Figures S1 and S2 show the actigraphy and questionnaire results under the control and active conditions by intervention device type (light tables, light trays, ambient room lighting). Although there were insufficient data within each device type to perform statistical comparisons (e.g., actigraphy and questionnaire data were available for only a single participant exposed to the light tray, active condition), the figures demonstrate the relatively small variability among intervention types (most importantly within the active intervention condition). Due to the small sample sizes and relatively small variability, we combined data from the 3 intervention types for each condition to perform statistical comparisons between the conditions.

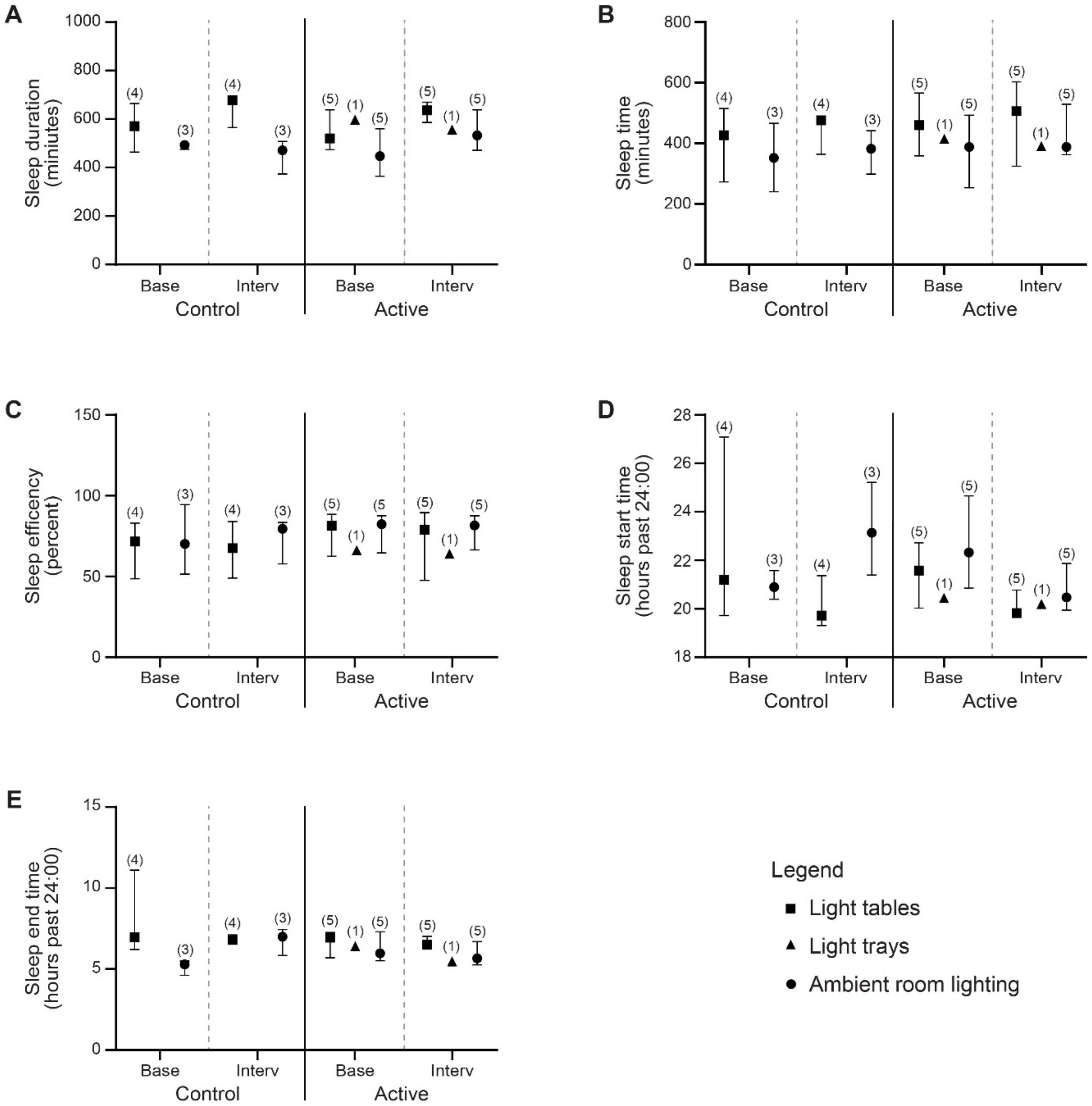

Supplementary Figure S1. Actigraphy results under the control and active conditions by intervention device type (light tables, light trays, ambient room lighting).

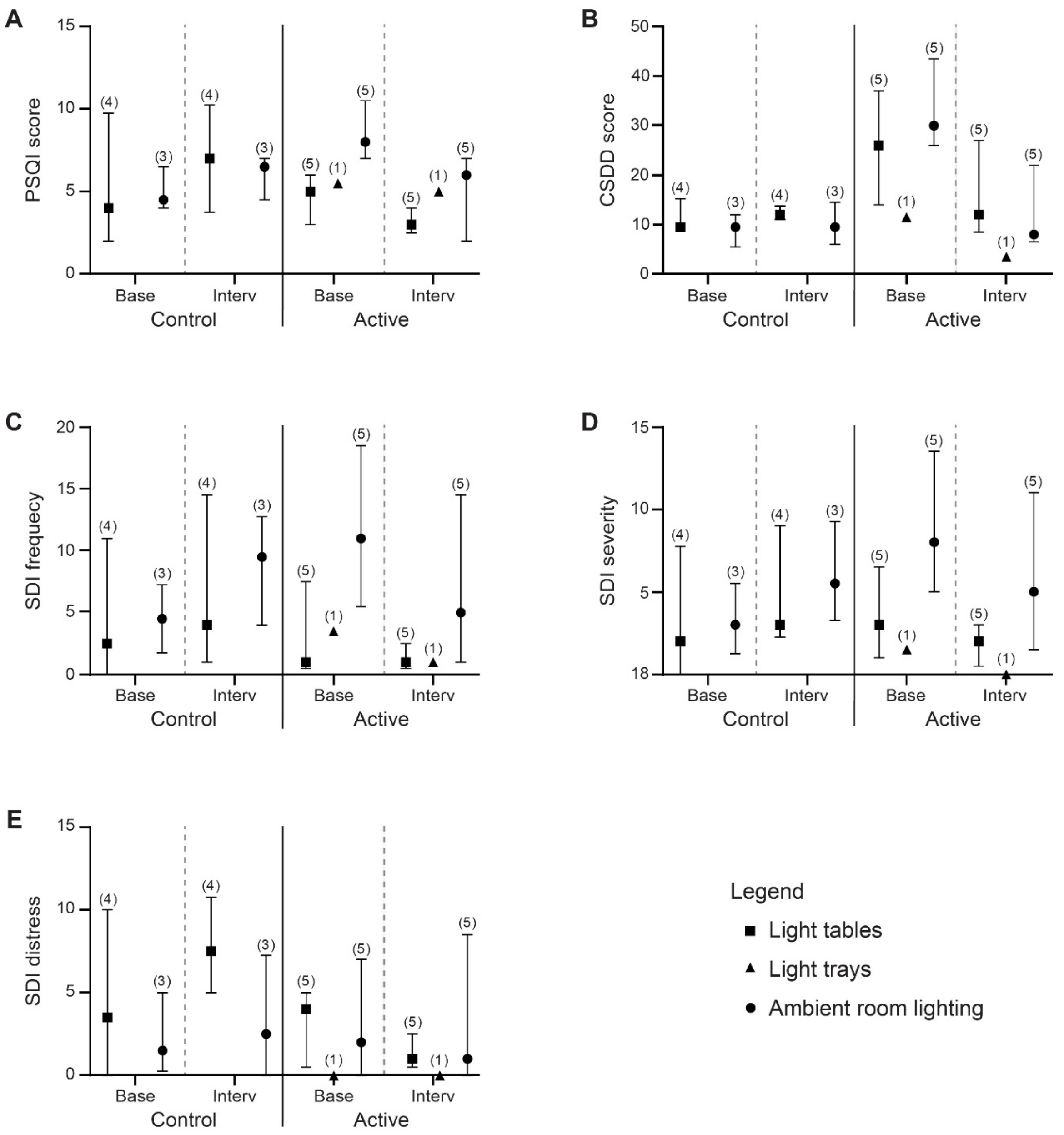

Supplementary Figure S2. Questionnaire results under the control and active conditions by intervention device type (light tables, light trays, ambient room lighting).
